# Highly Upregulated Expression of CD36 and MSR1 in Circulating Monocytes of Patients with Acute Coronary Syndromes

**DOI:** 10.1007/s10930-012-9431-8

**Published:** 2012-07-05

**Authors:** Michal Piechota, Anna Banaszewska, Joanna Dudziak, Marek Slomczynski, Robert Plewa

**Affiliations:** 1 Department of Animal Physiology and Development, Faculty of Biology, Adam Mickiewicz University, Umultowska 89 Str., 61-614 Poznan, Poland; 2 Department of Cardiology, Jozef Strus Municipal Hospital, Szwajcarska 3 Str., 61-285 Poznan, Poland

**Keywords:** Atorvastatin, Acute Coronary Syndromes, Monocytes, CD36, MSR1

## Abstract

Acute Coronary Syndromes (ACS) are a group of disorders caused by the significant reduction of circulation in coronary arteries. The most common reason of the dysfunction is a blood clot formed in place of plaque rupture. The role of scavenger receptors in development and progression of atherosclerosis has been confirmed in many animal experiments, however the knowledge about contribution of the receptors in the development of ACS symptoms in humans still remains insufficient. The aim of this work was to define the expression of two scavenger receptors: CD36 and MSR1 in monocytes of patients with ACS after the onset of symptoms and after the 6 months of treatment. The analysis of *CD36* and *MSR1* expression was carried out with the use of real-time PCR and flow cytometry. Analyses of lipid and glucose concentration in blood and the level of inflammatory markers in plasma were performed additionally for all ACS patients. All data obtained during the research were analyzed using statistical tests, such as Mann Whitney test, Wilcoxon test, or correlation. In all patients with symptoms of ACS the amount of *CD36* and *MSR1* mRNA in circulating monocytes, as well as the density of both receptors on the cells surface was significantly higher. Re-analysis of subjects after 6 months of treatment, showed a significant decrease in the CD36 and MSR1 expression in all patients who received atorvastatin. The results of presented studies demonstrate that both investigated receptors are involved in the development and/or progression of ACS.

## Introduction

Acute Coronary Syndromes (ACS) are a group of disorders that can be caused by a significant reduction of blood flow in coronary arteries due to narrowing or blockage of the vessels. The most common reason of the vessel dysfunction is the development of atherosclerotic lesions and a blood clot formed in place of plaque rupture [2, 39]. Oxidised lipoproteins (oxLDL) are the major factor responsible for the initiation and the acceleration of atherosclerosis. The processes activated by oxLDL are: endothelial dysfunction, expression of adhesion molecules, synthesis of chemoattractants and proinflammatory cytokines, or activation of monocytes and smooth muscle cells to migration into the area of inflammation [4, 6, 10, 21]. Monocyte-derived macrophages are able to bind and accumulate of modified lipoproteins through the scavenger receptors-mediated endocytosis [14–16, 34]. That is the main mechanism responsible for the formation of atherosclerotic plaque, because macrophages filled with cholesterol esters transform into foam cells and form the lipid core of the plaque [3, 29]. Scavenger receptors family is a large group of proteins, divided into six classes (from A to F). All proteins are able to recognize a large variety of ligands, such as: oxidized or acetylated LDL lipoproteins, polyanions, or apoptotic cells [11, 37]. The CD36, a type B scavenger receptor, and the MSR1, a type A, are the main receptors responsible for the uptake of modified lipproteins by macrophages [12, 33, 42]. Both proteins are able to remove 70–90 % of modified LDLs, however CD36 is a receptor responsible for oxLDL degradation, while the MSR1 is mainly involved in the uptake of acetylated lipoproteins (acLDL) [28].

The most common drugs used in the treatment of hypercholesterolemia and the reduction of the risk of cardio-vascular events in patients with ischemic heart disease are statins. The main therapeutic action of the drugs is the lowering of endogenous cholesterol synthesis through the inhibition of 3-hydroxy-3-methylglutaryl coenzyme A reductase (HMG-CoA reductase)—the key enzyme of the process [7, 27]. Recent studies on the lipid lowering drugs showed also a wide range of pleiotropic activities of statins that include enhancing of endothelial differentiation and stabilization [5, 38], reduction of the expression of adhesion molecule on the endothelial surface, suppression of monocyte/macrophage activation and differentiation [9, 18], inhibition of smooth muscle cell migration and proliferation, or stabilisation of atherosclerotic plaque [19, 25, 36, 43]. A very important object in the research on additional activities of statins is the influence of the treatment on the uptake of modified lipoproteins by macrophages. Statins are able to reduce the level of LDL modifications due to their lipid-lowering activity and through the reduction of reactive oxygen species (ROS) concentration in plasma [1, 17, 23, 26]. However the influence of the drugs on the expression of scavenger receptors, the process of oxLDL accumulation and the foam cell formation remains unclear and is constantly investigated [20, 30].

The aim of this work was to investigate the expression of CD36 and MSR1 scavenger receptors in monocytes isolated directly from fresh anticoagulated blood of patients with ACS. The purpose of the first part was to investigate alterations in the receptors expression in patients after the onset of the ACS symptoms. In the second part the experiments were repeated after the 6 months of atorvastatin therapy to determine the effect of treatment on the *CD36* and *MSR1* expression in monocytes.

## Materials and Methods

### Investigated and Control Group

Presented studies were carried out on the group of 100 patients with atherosclerosis, hospitalized for the onset of symptoms of ACS, defined as acute myocardial infarction with ST elevation (STEMI) or without (NSTEMI). The whole group consisted of 32 females and 68 males, aged from 33 to 78 years old (average 64). In 66 individuals additional disorders were diagnosed also, such as diabetes mellitus type 2 (NIDDM)—34 cases, arterial hypertension (HTN)—56 cases, or obesity—14 cases. Before hospitalization none of the patients received statins and after the onset of ACS symptoms in all analyzed cases the treatment with statins was applied. The atorvastatin was received by 20 patients from the investigated group, in a dose of 40 mg per day. 40 control subjects were also investigated, 20 females and 20 males, ranging from 26 to 54 years (average 37). The main criteria for selecting the control group were normal laboratory findings, such as the lipid concentration in plasma, blood glucose level, C-reactive protein (CRP) level in plasma and good general health.

The material in the investigations presented was fresh patient’s anticoagulated blood taken at the latest 24 h after the onset of ACS symptoms, and after 6 months of therapy with atorvastatin. The blood was used to extract DNA and RNA, to implement the molecular analyses, such as real-time PCR or flow cytometry, and to perform the basic laboratory tests, such as blood lipid concentration, blood glucose level or the level of C-reactive protein (CRP) in plasma.

### Monocyte Isolation

The first step of the analysis was to separate the monocytes from other blood cells by density-gradient centrifugation in Ficoll-Pague PLUS (GE Healthcare) and magnetic separation with Dynabeads^®^ CD14 (Invitrogen). The isolation was performed according to the manufacturers’ protocol. Peripheral blood mononuclear cells obtained after ficoll centrifugation were incubated with the superparamagnetic polystyrene beads coated with monoclonal anti-CD14 antibodies and next the CD14^+^ cells (monocytes) were separated by placing the sample in a strong magnetic field.

### RNA Extraction and cDNA Synthesis

Purified monocytes were used directly to the total RNA isolation using TRI REAGENT^®^ BD (Sigma Aldrich). The procedure was performed according to the manufacturer’ procedure which was designed for RNA extraction from blood cells, based on the single-step RNA isolation reported by Chomczynski and Sacchi [8]. After the extraction the concentration of RNA in samples was estimated, by micro-volume UV-Vis spectroscopy (NanoDrop; Thermo Scientific). The amount containing 500 ng of RNA was used to reverse transcription PCR (RT-PCR) reaction. The synthesis of complementary DNA (cDNA) was performed in a total volume of 20 μl, using oligo(dT)_18_ primer (100 ng/μl), RNase free, DEPC treated water and RevertAid™ M-MuLV Reverse Transcriptase (Fermentas).

### Real-Time PCR Reaction

The real-time PCR reaction was used to determine the level of CD36 and MSR1 transcripts in monocytes derived from peripheral blood. The amplification was performed with specific primers designed using oligo 6.65 software (Rychlik and Rhoads 1989–2002). As a reference for the analysis of *CD36* and *MSR1* expression was used gene of porphobilinogen deaminase (*PBGD*). The real time amplification (DyNAmo™ HS SYBR^®^ Green qPCR Kit, Finnzymes) was carried out separately for the investigated, CD36 and MSR1 genes and for the *PBGD*, in a final reaction volume of 20 μl. The conditions of the reaction were: hold at 95 °C for 15 min, denaturation at 95 °C for 10 s, annealing at 56 °C for 15 s and elongation at 75 °C for 20 s, for 45 cycles. The final elongation was performed at 72 °C for 10 min and preceded by a process of the appointing of melting curve, carried out at 72–95 °C for 20 min. The specificity of the real-time PCR reaction was based on the melting curve. All analyzes were performed using the RotorGene 6000 software (Corbett Research).

### Flow Cytometry Analysis

Flow cytometry was used to analyze the density of CD36 and MSR1 receptors on the surface of patient’s monocytes. The process was carried out using 100 μl of anticoagulated peripheral blood and monoclonal antibodies connected with specific days. The antibodies targeted three different antigens: CD36 (anti-Human CD36 FITC, Beckman Coulter), MSR1 (anti-Human MSR1 PE, R&D Systems) and CD14 (anti-Human CD14 PC5, Beckman Coulter). The first two were used to determine the density of investigated receptors, while the latter enabled the identification of monocyte population from all leukocytes. All samples were incubated for half an hour with antibodies, under conditions defined by manufacturers, and then prepared for analysis through lysis of erythrocytes (formic acid), leukocytes stabilization (PBS buffer) and paraformaldehyde fixation, using IMMUNOPREP™ Kit (Beckman Coulter). All analyses were performed using the Cell Lab Quanta flow cytometer (Beckman Coulter).

### Quantitative Determination of Oxidized LDL in Plasma

For all patients the analysis of oxidized LDLs concentration in plasma with the use of enzyme immunoassay: Oxidized LDL ELISA (Biomedica) was also performed. In the assay polyclonal anti-oxLDL antibodies are absorbed into microwells. Modified lipoproteins presented in sample or in standard bound to the antibodies and then HRPO(horseradish peroxidase)-conjugated monoclonal anti-oxLDL antibody bound to modified LDL captured by the first antibody. In the next step to the reaction, the substrate for the enzyme was added and the amount of colored product was measured. The colored product was formed in proportion to the amount of oxLDL in investigated plasma or standard and it was determined by the measurement of absorbance at 450 nm, with the use of microwell strip reader: Multimode Detection System DTX 880 (Beckman Coulter).

### Statistical Analysis

For all data obtained during the research statistical analyses were performed using GraphPad Prism 5 software. The comparison of results for patients versus control group was performed with the use of Mann Whitney test, while the data obtained for patients before and after atorvastatin treatment were analyzed with the use of Wilcoxon test. The values of the oxLDL concentration in plasma were used to define the correlation between the elevated concentration of modified lipoproteins in plasma and the level of CD36 and MSR1 expression in monocytes of patients after the onset of ACS symptoms. The value of *p* < 0.05 was considered to be statistically significant.

## Results

The results were divided into three parts. The first analysis concerned the expression level of *CD36* and *MSR1* in patients with ACS and the relations between the receptors expression and oxLDLs concentration in plasma. The second part was targeted on investigation of the alterations in the CD36 and MSR1 genes expression after 6 months of atorvastatin treatment. Additionally based on the values of laboratory analysis the third part was established, which was the effectiveness of the therapy in the lipid and inflammation lowering.

At the level of mRNA, the expression of investigated genes was defined as a relative amount of *CD36* and *MSR1* transcripts normalized to a reference PBGD gene and relative to the healthy subjects, defined as 2^−ΔΔCT^. At the protein level the density of the scavenger receptors on the monocte surface was determined by the level of specific fluorescence emitted by stained antibodies connected with investigated antigens, and defined as FSD (Fluorescent Surface Density) parameter.

### The Level of CD36 and MSR1 Expression was Significantly Increased in All ACS Patients

**Fig. 1:**
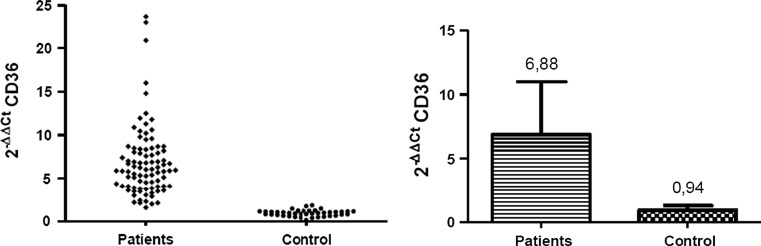
The results of quantitative analysis of *CD36* transcript level. The *graph* on the *left* presents the mean value of relative expression of CD36 gene in each analysed case (*n* = 3). On the *right* are presented the relative amounts of *CD36* transcript in the patients with ACS and in the control group. Results are expressed as mean 2^−ΔΔCT^ value ± SD. *p* < 0.0001

The quantitative real-time PCR analysis of the CD36 gene expression was performed for every patient and healthy individual classified to the investigation. The expression of *CD36* was at least sixfold higher in all analyzed cases of ACS in comparison to healthy control. The mean amount of CD36 gene transcripts in monocytes was 6.88 ± 4.12 in the patients, while in the control group it was 0.94 ± 0.36 (Fig. 1). All results were statistically significant (*p* < 0.0001).

**Fig. 2:**
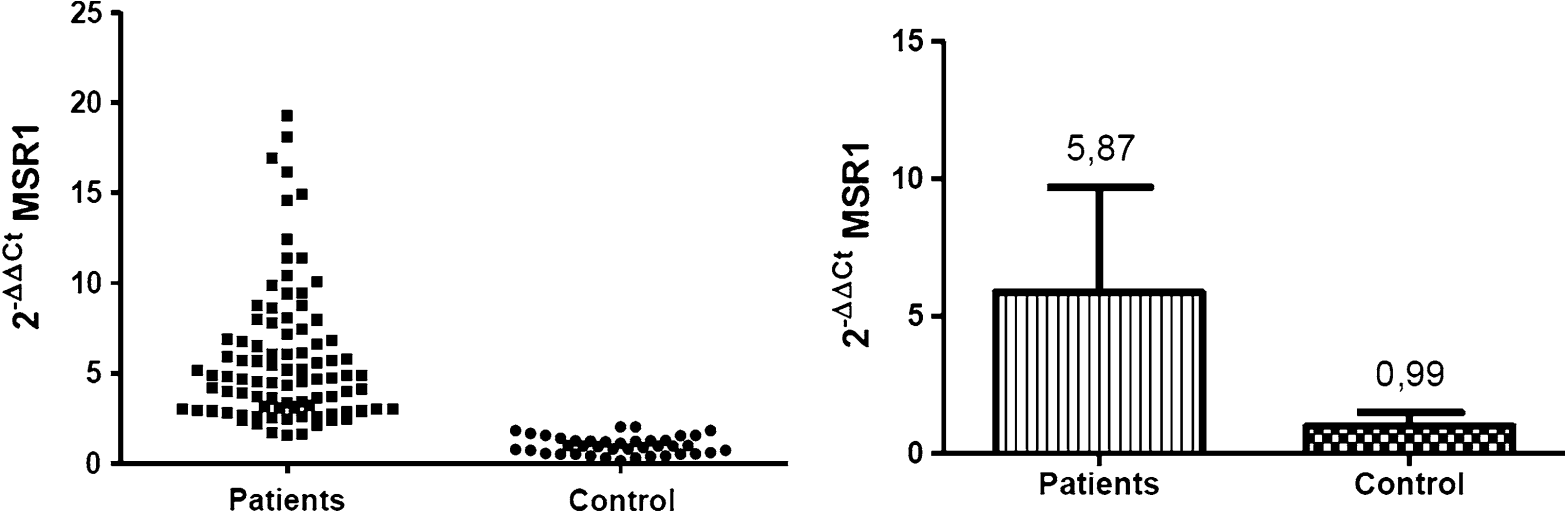
The results of quantitative analysis of *MSR1* transcript level. The *graph* on the *left* presents the mean value of relative expression of MSR1 gene in each analysed case (*n* = 3). On the *right* are presented the relative amounts of *MSR1* transcript in the patients with ACS and in the control group. Results are expressed as mean 2^−ΔΔCT^ value ± SD. *p* < 0.0001

Analogously analysis were performed for the MSR1 gene. The level of MSR1 gene expression in monocytes of patients after the onset of ACS symptoms was at least fivefold higher than in healthy individuals. The mean amount of *MSR1* transcripts in the investigated group and in control group was 5.87 ± 3.83 and 0.99 ± 0.5 respectively (Fig. 2). All results were statistically significant (*p* < 0.0001).

Another step was the flow cytometric analysis. Based on the FSD parameter value, both for the investigated and control group was defined the density of the scavenger receptors on the monocyte surface. For CD36 the FSD parameter was determined on the basis of the level of fluorescein isothiocyanate (FITC) fluorescence, while the MSR1 FSD parameter was defined on the basis of the level of Phycoerythrin (PE) fluorescence.

**Fig. 3:**
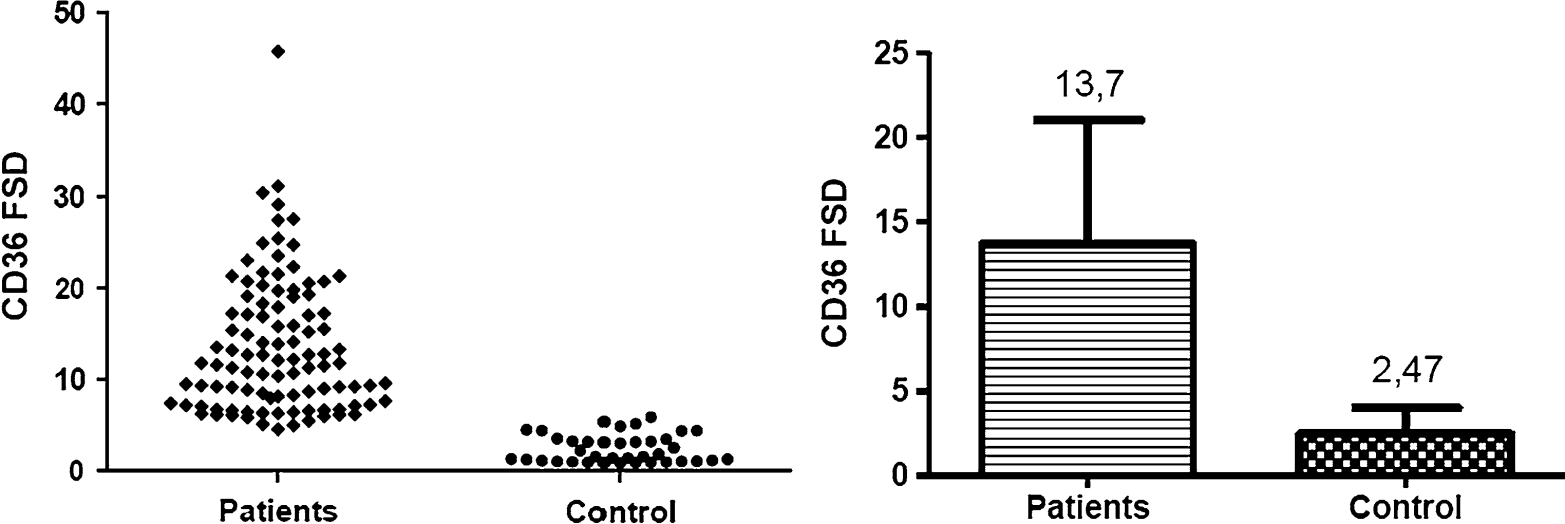
The results of flow cytometric analysis of CD36 density on the monocyte surface. The *graph* on the *left* presents the mean value of FSD parameter in each analysed case (*n* = 3). On the *right* are presented the mean values of CD36 density on monocyte surface in the patients with ACS and in the control group, expressed as mean value of FSD parameter ± SD. *p* < 0.0001

**Fig. 4:**
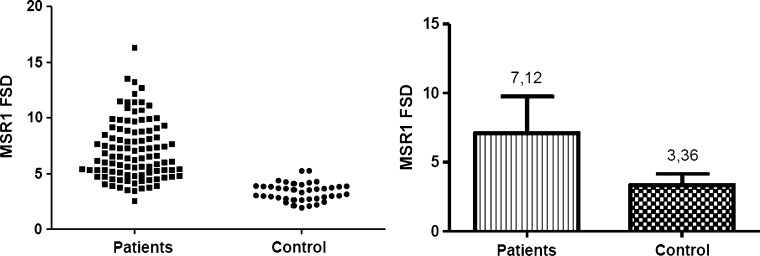
The results of flow cytometric analysis of MSR1 density on the monocyte surface. The *graph* on the *left* presents the mean value of FSD parameter in each analysed case (*n* = 3). On the *right* are presented the mean values of MSR1 density on monocyte surface in the patients with ACS and in the control group, expressed as mean value of FSD parameter ± SD. *p* < 0.0001

In all analyzed patients the density of both receptors on the monocyte surface was significantly higher than in healthy subjects. The mean values of CD36 FSD was 13.7 ± 7.35 and 2.47 ± 1.54 in the patients and in the control group respectively (Fig. 3), while the FSD parameter for MSR was 7.12 ± 2.65 in the patients and 3.36 ± 0.8 in the control subjects (Fig. 4). All results were statistically significant (*p* < 0.0001).

### The Density of Both Receptors on the Surface of Monocytes was Positively Correlated with the Concentration of oxLDL in Plasma

**Fig. 5:**
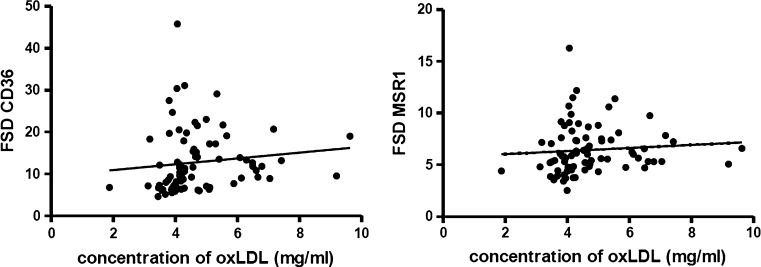
Correlation between the concentration of oxLDL in plasma and the density of investigated receptors on the surface of monocytes. The *graph* on the *left* shows the results for CD36 (r = 0.35; *p* = 0.0017), while the *graph* on the *right* presents the correlation for MSR1 (r = 0.26; *p* = 0.02)

Performed statistical analyses showed that there was no statistically significant correlation between the concentration of oxLDLs and the level of *CD36* or *MSR1* transcripts. However the analysis of data obtained from flow cytometric measurements showed that there was a positive correlation between the elevated concentration of oxidized lipoproteins in plasma and increased density of both investigated receptors on the monocyte surface (Fig. 5).

### Atorvastatin Reduced the Expression of CD36 and MSR1 in All Patients

**Fig. 6:**
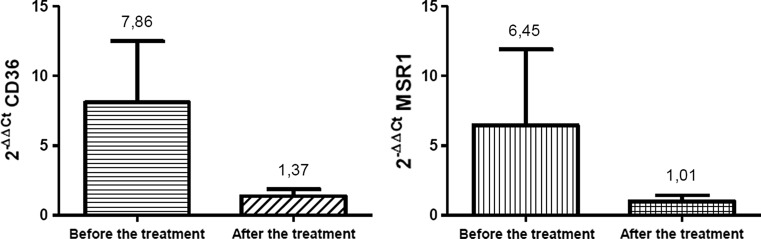
The effects of atorvastatin treatment on the *CD36* and *MSR1* expression. Results are expressed as mean 2^−ΔΔCT^ value ± SD. The p value is respectively: *p* < 0.0001 and *p* = 0.0017

The second part of our investigation was the re-analysis of the scavenger receptors expression in all patients, after 6 months of atorvastatin treatment. Obtained results showed a significant decrease in the expression of CD36 and MSR1 in all cases. The level of *CD36* transcript and *MSR1* transcript was reduced down approximately to levels observed in control group. The expression of CD36 was redused from 7.86 ± 4.43 to 1.37 ± 0.5, while the expression of MSR1 was 6.45 ± 5.66 before the treatment and 1.01 ± 0.42 after the atorvastatin therapy (Fig. 6).

**Fig. 7:**
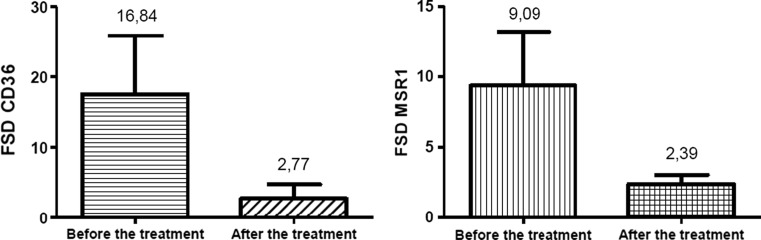
The effects of atorvastatin treatment on the density of CD36 and MSR1 on the monocyte surface. Results are expressed as Mean FSD parameter value ± SD. The *p* value is respectively: *p* < 0.0001 and *p* = 0.002

Also the comparison of the flow cytometric results showed the reduction of receptors density on the surface of monocytes in all patients who received atorvastatin. The density of CD36 after the onset of ACS symptoms was 16.84 ± 8.53 while after the treatment it was reduced to the value of 2.77 ± 2.02. Similarly the density of MSR1 was decreased from 9.09 ± 3.24 to 2.39 ± 0.66 (Fig. 7). All results were statistically significant (*p* < 0.05).

### The Values of Laboratory Tests Demonstrated the Effectives of Atorvastatin Treatment

Analysis of blood lipid and glucose concentration and the level of CRP in plasma were performed for all patients after the onset of ACS symptoms and after the treatment. The mean values of obtained data are presented in the table below along with standard laboratory values for the performed tests (Table 1.)

**Table 1:** The values of laboratory blood tests for patients with ACS, before and after atorvastatin therapy

	Before atorvastatin	After atorvastatin	Laboratory standards for healthy people
Total cholesterol (mmol/l)	5.67 ± 1.46	4.17 ± 0.8	3.62–5.2
LDL cholesterol (mmol/l)	3.61 ± 1.34	2.2 ± 0.76	2.6–3.3
HDL cholesterol (mmol/l)	1.34 ± 0.4	1.4 ± 0.36	1.68–2.5
Triglicerydes (mmol/l)	1.59 ± 0.84	1.33 ± 0.5	0.6–1.7
Glucose (mmol/l)	8.06 ± 3.15	5.65 ± 0.8	3.9–6.1
CRP (mg/dl)	74.15 ± 31.53	1.97 ± 1.88	≤3

The data is presented as the mean values along with standard values for the performed tests

The six-month treatment resulted in significant (*p* < 0.05) reduction of total and LDL cholesterol concentration in all patients who received atorvastatin. Similar results were obtained for the blood glucose concentration and the level of inflammatory factors (CRP) in plasma, while the HDL level and the serum triglycerides concentration wasn’t significantly changed.

## Discussion

The described research had the purpose of analyzing CD36 and MSR1 scavenger receptors expression in monocytes of patients with ACS. The topic still remains important because despite the fact that significant role of scavenger receptors in the pathogenesis of atherosclerosis has been confirmed in many experiments [12, 14–16, 28, 33, 42], carried out mostly in animal models, the knowledge about contribution of the receptors in the development of ACS symptoms in humans still remains insufficient.

Data obtained in our investigations demonstrate that in patients with ACS the expression of CD36 and MSR1 is significantly increased already in the circulating monocytes. This fact is in contradiction with some of the previous hypotheses that the expression of scavenger receptors is significantly increased especially during the evolution of monocytes to macrophages [20]. In patients with ACS up-regulated expression of investigated receptors in circulating monocytes can be strongly associated with the late stage of atherosclerosis, because the onset of acute symptoms is characterized by increased activity of monocytes and macrophages, as well as very high level of inflammation in the organism [4, 21, 39]. This hypothesis was confirmed in presented research through the analysis of the C-reactive protein concentration in plasma, which showed that in patients with acute coronary syndrome the level of CRP was even 100 fold higher than the accepted standards for healthy people. Another step in our investigation about the role of scavenger receptors in the development and progression of atherosclerosis was the analysis of correlation between the level of oxidized lipoproteins in plasma and the expression of investigated receptors on the surface of monocytes. For all patients with ACS, measurements of oxLDL concentration was performed and obtained data were compared with the results of CD36 and MSR1 expression in monocytes. For both receptors the statistical analyses showed positive correlation between the density of receptors on monocyte surface and the elevated concentration of modified lipoproteins in plasma. These findings seem to confirm the relationship between the elevated level of CD36 and MSR1 expression and the cardio-vascular disorders, however low correlation coefficient in both cases (r_CD36_ = 0.35 and r_MSR1_ = 0.25) and lack of correlation between the level of *CD36*/*MSR1* mRNA and the oxLDL concentration suggest that this topic requires further investigation. Some findings also suggest that the level of MSR1 gene expression increases only during the monocyte to macrophages differentiation [22, 31], however the results of our study showed that it may be increased already in circulating monocytes, particularly in patients after acute myocardial infarction. For this reason our findings suggest that the analysis of scavenger receptors can be useful in the diagnostics of the cardio-vascular risk in patients, however this issue need further investigations.

In the second part of the study, our goal was to demonstrate the effect of atorvastatin therapy on the level of CD36 and MSR1 expression. The effectiveness of the treatment was confirmed through the laboratory tests, because in all patients who received atorvastatin the values of LDL, glucose and CRP concentration in blood returned to the level suitable for healthy individuals. Moreover, performed analyses showed that the treatment didn’t significantly influence the level of blood HDL level, and these results are characteristic for the atorvastatin activity [25]. The influence of statins on the monocytes/macrophages activation, oxLDL accumulation and formation of foam cells still remains valid, because the results obtained so far come mainly from studies carried out in the in vitro conditions, and available data is often contradictory and ambiguous. For example the treatment with pravastatin didn’t show effects on the uptake of modified lipoproteins by macrophages derived from hipercholesterolemic subjects [27]. It is noteworthy also that in rats with metabolic disorders the cerivastatin therapy led to increased expression of CD36 receptors [32]. Oppositely, the findings obtained from in vitro studies on the effects of fluvastatin, simvastatin or lovastatin therapy on the level of scavenger receptors expression demonstrated that the drugs significantly decreased the expression of LOX-1, SRA-I, SRA-II and CD36 [13, 24, 35, 40, 41]. In our investigations, the atorvastatin therapy had an impact on the level of CD36 and MSR1 expression in circulating monocytes. In all patients the level of *CD36* and *MSR1* mRNAs, and the density of the receptors on the surface of monocytes was reduced down from three to sixfold, to levels observed in the control group. The results confirmed findings obtained for in vivo investigations of monocytes of patients with Type 2 Diabetes [30] and are in agreement with similar investigations carried out on monocyte-derived macrophages of patients with hypercholesterolemia [20]. Obtained results suggest that atorvastatin reduces the expression of investigated receptors, however the treatment also had an influence on other factors that may be associated with the expression of CD36 and MSR1, thus, a direct mechanism of atorvastatin action requires further research.

## Conclusion

The results of presented studies demonstrate that in patients with the onset of symptoms of ACS highly up-regulated expression of CD36 and MSR1 in the circulating monocytes is connected with the progression of the coronary disease, and confirm the role of scavenger receptors in the development of cardio-vascular disorders. The findings also suggest that atorvastatin significantly reduces the expression of scavenger receptors, however due to the wide range of pleiotropic effects of statins the direct mechanism of the drug activity remains unclear.

## Abbreviations

HMG-CoA: 3-Hydroxy-3-methylglutaryl-CoA
CD36: Macrophage scavenger receptor class B
MSR1: Macrophage scavenger receptor class A
LDL: Low density lipoprotein
oxLDL: Oxidized low density lipoprotein
ROS: Reactive oxygen species
acLDL: Acetylated low density lipoprotein
ACS: Acute Coronary Syndromes
STEMI: Acute myocardial infarction with ST elevation
NSTEMI: Acute myocardial infarction without ST elevation
PBGD: Porphobilinogen deaminase
HRPO: Horseradish peroxidase
FSD: Fluorescent surface density
CRP: C-reactive protein
